# Current challenges and emerging solutions in dentistry for treating medication-related osteonecrosis of the jaw (MRONJ): the state of the art in the era of personalized medicine

**DOI:** 10.31744/einstein_journal/2026RW2547

**Published:** 2026-08-14

**Authors:** Stéfany Barbosa Alves da Cruz, Mirela Caroline da Silva, Mileni Buzo-Souza, Gabriela Fulanetto Crestana, Fernanda Faot, Leticia Mello Bezinelli, Jamil Awad Shibli, Leonardo Perez Faverani

**Affiliations:** 1 Universidade de São Paulo Faculdade de Odontologia Department of Diagnosis and Surgery Araçatuba SP Brazil Division of Oral and Maxillofacial Surgery and Implantology, Department of Diagnosis and Surgery, Faculdade de Odontologia, Universidade de São Paulo, Araçatuba, SP, Brazil; 2 Universidade Estadual de Campinas Faculdade de Odontologia de Piracicaba Department of Oral Diagnosis Piracicaba SP Brazil Department of Oral Diagnosis, Faculdade de Odontologia de Piracicaba, Universidade Estadual de Campinas, Piracicaba, SP, Brazil.; 3 Hospital Israelita Albert Einstein Faculdade Israelita de Ciências da Saúde Albert Einstein São Paulo SP Brazil Faculdade Israelita de Ciências da Saúde Albert Einstein, Hospital Israelita Albert Einstein, São Paulo, SP, Brazil.; 4 Universidade Federal de Pelotas Faculdade de Odontologia Department of Restorative Dentistry Pelotas RS Brazil Department of Restorative Dentistry, Faculdade de Odontologia, Universidade Federal de Pelotas, Pelotas, RS, Brazil.; 5 Universidade de Guarulhos Dental Research Division Department of Periodontology Guarulhos SP Brazil Dental Research Division, Department of Periodontology, Universidade de Guarulhos, Guarulhos, SP, Brazil.; 6 Harvard School of Dental Medicine Division of Periodontology Department of Oral Medicine, Infection, and Immunity Boston MA USA Department of Oral Medicine, Infection, and Immunity, Division of Periodontology, Harvard School of Dental Medicine, Boston, MA USA.; 7 Research Unit Periodontology and Oral Microbiology KU Leuven, Department of Oral Health Sciences Leuven Belgium KU Leuven, Department of Oral Health Sciences, Research Unit Periodontology and Oral Microbiology (P&OM), Leuven, Belgium.

**Keywords:** Osteonecrosis, Jaw diseases, Biphosphonates, Denosumab, Bone remodeling

## Abstract

**Objective:**

Medication-related osteonecrosis of the jaw (MRONJ) is a serious complication associated with antiresorptive, and antiangiogenic therapies commonly used to treat osteoporosis, malignant bone tumors, and metastatic diseases. This review provides a comprehensive overview of the pathophysiology, clinical presentation, diagnosis, prevention, and management of MRONJ.

**Methods:**

This narrative review examined the prevalence of MRONJ and its clinical and radiological features, particularly those relevant to disease staging, and outlined current preventive and treatment strategies, aiming to improve patient outcomes and quality of life.

**Results:**

Patients with cancer receiving high-dose intravenous antiresorptive medications exhibited a higher incidence of MRONJ than that observed in patients treated for osteoporosis. Clinical staging systems, especially those developed by the American Association of Oral and Maxillofacial Surgeons, facilitate disease classification based on clinical and imaging findings, thereby guiding treatment selection and prognosis assessment. Preventive approaches, conservative management, surgical procedures, and adjunct therapies have been proposed to promote healing of both hard and soft tissues.

**Conclusion:**

Although emerging therapeutic options are promising, prevention and personalized treatment remain major challenges.

## INTRODUCTION

A major concern in maxillofacial bone remodeling is the disruption of osteoblastogenesis and osteoclastogenesis associated with various bone disorders, including osteoporosis, Paget's disease, hypovitaminosis D, multiple myeloma, osteosarcoma, and other malignant bone tumors. Antiresorptive drugs, particularly bisphosphonates or anti-receptor activator of nuclear factor kappa-B ligand (RANKL) monoclonal antibodies (denosumab), and antiangiogenic drugs are widely used in the management of these conditions.

Despite their well-established antiresorptive and antiangiogenic effects, these agents, depending on the dosage and treatment regimens, may be associated with adverse effects, most notably medication-related osteonecrosis of the jaw (MRONJ). Bisphosphonates, the most commonly used antiresorptive drugs, have a high affinity for hydroxyapatite crystals, resulting in prolonged retention within bone tissue.^(1)^ Under physiological conditions, bone remodeling involves osteoclastic resorption followed by osteoblastic bone matrix formation. However, when bisphosphonates accumulate in bone tissue, they are internalized by osteoclasts, inducing apoptosis and suppressing bone resorption.^(2,3)^ Consequently, osteolytic activity is reduced, contributing to alterations in bone remodeling in patients receiving treatment for systemic skeletal disorders such as those mentioned above. Therefore, considerable efforts have focused on restoring normal bone microarchitecture and bone density.^(4)^

Denosumab is a human monoclonal antibody that binds to the RANKL receptor activator, mimicking the inhibitory action of endogenous osteoprotegerin. By preventing the interaction between RANKL and its receptor (RANK) on osteoclasts, denosumab inhibits osteoclast differentiation, activation, and survival, ultimately reducing bone resorption.^(5)^

In this narrative review, the authors provide an in-depth discussion of the pathophysiological mechanisms underlying medications associated with MRONJ, with a particular emphasis on antiresorptive agents. We also review the epidemiology, clinical and imaging manifestations, and disease staging, as well as current prevention and treatment strategies, with the aim of improving patient outcomes and quality of life.

### Pathophysiology

The pathophysiology of MRONJ is linked to suppressed bone turnover. This effect is particularly relevant in the maxilla and mandible because these bones undergo high rates of physiological remodeling and are continually exposed to local challenges, including masticatory microtraumas, periodontal disease, periapical lesions, orthodontic tooth movements, pathological tooth migration, extractions, implant placement, and reconstructive procedures.^(6,7)^ When osteoclastic activity is reduced, damaged bone tissue cannot be adequately resorbed and replaced, resulting in the accumulation of non-viable bone tissue. Clinically, this process may manifest as non-repairable alveolar bone following tooth extractions or as bone sequestration, which is a bone fragment that detaches from the base.^(8)^

Inflammation and infection are linked to the development of jaw osteonecrosis; therefore, MRONJ should not be considered aseptic necrosis. Impaired bone remodeling limits the ability of bone tissue to produce an effective reparative response to cytokines and proinflammatory cells.^(7,9)^ Inflammation is often accompanied by bacterial colonization, particularly by anaerobic bacteria, which further amplifies the local inflammatory response and promotes tissue necrosis. Clinically, these processes commonly present with purulent exudate and pain. Accordingly, strict oral hygiene and regular dental follow-up should be emphasized before and throughout antiresorptive therapy.^(10,11)^

Recent evidence suggests that the association between tooth extraction and MRONJ development may be driven less by the surgical procedure itself than by the underlying oral inflammatory and infectious conditions that necessitate extraction, particularly periodontal and periapical diseases.^(12,13)^ Accordingly, tooth extraction may frequently serve primarily as a surrogate marker for pre-existing chronic infection rather than an independent precipitating event.^(13)^ Persistent periodontal inflammation may promote microbial dysbiosis, amplify the local inflammatory response, impair tissue repair, and disrupt bone remodeling, thereby creating a biological environment that favors MRONJ development in susceptible patients receiving antiresorptive therapy.^(14)^ Consequently, comprehensive periodontal evaluation and timely management of oral inflammatory conditions should be considered integral components of contemporary MRONJ prevention strategies.^(7,12,13,15)^ Another factor is the significant reduction in local vascularization induced by bisphosphonates.^(14,16)^ Reduced vascularization prevents the substrates necessary for bone repair from reaching the stipulated site and significantly compromises tissue perfusion necessary for the cytoplasm and extracellular matrix, ultimately leading to cell death.^(17)^

However, oral damage related to antiresorptive therapy is not restricted to bone tissue. Adjacent soft tissues, such as the gingiva and alveolar mucosa, are affected, either through bacterial toxins, activated inflammatory cells, and cytokines or impaired angiogenesis.^(9)^ Consequently, tissue lysis and gradual soft tissue destruction occur, accelerating the exposure of necrotic bone. Despite receiving comparatively limited attention in the literature, the loss of these tissues is of considerable clinical concern because extensive bone exposure may preclude complete tissue closure owing to insufficient residual gingiva or mucosal tissue.^(2)^

In addition to the established mechanisms underlying MRONJ development, patients with systemic diseases such as advanced osteoporosis, rheumatic disorders, or malignancies with skeletal metastases, who are treated with antiresorptive or antiangiogenic drugs, often exhibit immune dysfunction that may also directly contribute to the development of this complication.^(7,18-21)^

Emerging evidence suggests that immune dysregulation plays a more prominent role than previously assumed. Experimental and translational studies have demonstrated altered macrophage polarization, impaired neutrophil function, and increased proinflammatory cytokine expression in MRONJ lesions^(22)^ indicating that host immune response may modulate both disease onset and persistence.

Although suppression of osteoclast-mediated bone remodeling is widely recognized as a central mechanism,^(2,3,23)^ accumulating evidence suggests that MRONJ cannot be explained solely by reduced bone turnover. Experimental and clinical data indicate that soft tissue impairment,^(9)^ altered vascularization,^(14-17)^ immune dysregulation,^(22)^ and microbial colonization^(10,11)^ interact synergistically to promote disease development. Rodent models have demonstrated MRONJ-like lesions only when systemic drug exposure was combined with local trauma or infection,^(18)^ supporting the concept that suppression of bone remodeling alone is insufficient to trigger the disease.

### Incidence

Most systematic reviews have reported that MRONJ predominantly affects female patients, most commonly those older than 50 years, with alendronate and zoledronate being the most prescribed antiresorptive medications.^(19-21)^

Despite this epidemiological profile, the literature indicates that the most advanced cases of MRONJ occur in patients with cancer, likely because of the administration of higher doses of antiresorptive drugs, in addition to the immunodeficiency associated with tumors and their treatment.^(22)^ Overall, studies suggest that the risk of MRONJ is approximately 5% among patients with cancer, compared to 0.05% among patients with osteoporosis.^(23-25)^

Regarding the relationship between drug exposure and MRONJ risk, among patients with cancer receiving zoledronate, the risk of MRONJ ranges from 0% to 18%, with an overall average incidence of approximately 5%. This wide variation is primarily attributable to the differences in follow-up duration across retrospective studies.^(23-25)^ Although RANKL inhibitors do not bind to bone tissue in the same way as bisphosphonates, and their effects diminish approximately 6 months after discontinuation, studies have reported a risk of MRONJ ranging from 0% to 6.9%, with most reporting rates of approximately 5%, comparable to those reported for zoledronate.^(26,27)^

Among patients with osteoporosis treated with bisphosphonates, the risk of MRONJ ranges from 0.02% to 0.05%, with the lowest risk reported in patients receiving annual intravenous zoledronate (0.02%). Conversely, patients receiving oral bisphosphonates, typically administered weekly, have an estimated MRONJ risk of 0.05%.^(28,29)^ Denosumab is associated with an even greater risk of MRONJ than that with bisphosphonates, with an estimated MRONJ incidence of 0.3% among treated patients.^(30)^

Owing to the long half-life of these medications and their interaction with bone tissue, the risk of developing MRONJ increases with prolonged use. Henry et al. reported that patients with cancer treated with denosumab or zoledronate had a markedly increased risk of developing MRONJ over 3 years.^(31)^

Recent literature has emphasized the importance of classifying patients receiving antiresorptive or antiangiogenic therapies into high-dose and low-dose groups, particularly when evaluating MRONJ risk and developing preventive and therapeutic strategies.^(7,23-32)^ Generally, patients with malignant diseases receive high-dose regimens administered at shorter intervals, whereas patients with osteometabolic conditions, such as osteoporosis, typically receive lower cumulative doses. This distinction may substantially influence MRONJ incidence, disease severity, prognosis, and treatment outcomes.^(23,24,27,30,31)^

Furthermore, the adoption of high-dose and low-dose classification models may facilitate the interpretation of clinical studies and improve the comparability of findings across different patient populations. Therefore, stratification based on drug exposure profiles should be considered in both clinical practice and future studies on MRONJ risk assessment and management.^(7,21,32,33)^

Although medication-related variables, including drug type, administration route, cumulative dose, and treatment duration, remain central to MRONJ risk assessment,^(7,21)^ current evidence indicates that MRONJ development is multifactorial.^(33-35)^ Recent studies and international papers have emphasized that MRONJ risk is significantly influenced by systemic and local factors. Systemic factors including malignant disease, chemotherapy, corticosteroid therapy, diabetes mellitus, smoking, immunosuppression, advanced age, and other comorbidities may compromise immune function, vascularization, and tissue repair.^(22,33,34)^ Local factors include periodontal and periapical diseases, poor oral hygiene, dentoalveolar surgical procedures, implant-related complications, prosthesis-related trauma, and chronic oral infections.^(6,8,10,35)^

Organizations such as the Italian Society of Oral Pathology and Medicine and the Italian Society of Maxillofacial Surgery (SIPMO-SICMF) have proposed broader risk assessment models that integrate medication-related, systemic, and local variables into an individualized patient assessment.^(33)^ This approach may help develop more effective preventive strategies, more accurate risk stratification, and improved treatment planning for patients receiving antiresorptive or antiangiogenic therapies.^(7,8,21,33,35)^

Reported incidence rates vary considerably with study design, follow-up duration, cumulative dose, and diagnostic criteria.^(23-27,29)^ Randomized controlled trials in oncology populations^(23-26)^ provide more reliable incidence estimates than those reported by retrospective case series; however, even phase 3 trials may underestimate late-onset cases because of limited follow-up. Furthermore, long-term extension studies of denosumab^(30)^ and delayed skeletal event analyses^(31)^ suggest that cumulative exposure significantly influences MRONJ risk over time.^(36)^ Therefore, reported incidence rates should be interpreted within their clinical context rather than as absolute measures.

### Clinical and imaging stages

Medication-related osteonecrosis of the jaw staging is used to identify and treat patients based on the clinical and imaging features of the disease.^(37)^ It also allows patient stratification to estimate prognosis and evaluate treatment outcomes. Early identification of clinical or imaging abnormalities, even before bone exposure occurs, is essential to prevent disease development in at-risk patients, including asymptomatic patients receiving intravenous or oral antiresorptive agents or even antiangiogenic therapy.^(7)^

The American Association of Oral and Maxillofacial Surgeons has recommended a staging system since 2009, which was subsequently revised in 2014 and updated in 2022.^(38,39)^

Stage 0, referred to as the unexposed bone variant, is characterized by the absence of clinically exposed necrotic bone. However, patients may present with unexplained pain, including pain radiating to the temporomandibular joint region or maxillary sinus region, without evidence of odontogenic disease or maxillary sinus mucosal inflammation. Radiographic findings may include trabecular bone alterations and the absence of normal bone healing within post-extraction sockets. This stage may also be observed in patients previously treated for more advanced stages who no longer exhibit clinical evidence of exposed bone.^(38,39)^

Stage 1 is characterized by exposed necrotic bone or a fistula communicating with bone. Lesions may be asymptomatic, with no clinical evidence of inflammation or infection. Notably, radiographic changes remain confined to the alveolar bone, similar to stage 0.^(38,39)^

Stage 2 is characterized by exposed necrotic bone or a fistula associated with evidence of inflammation or infection. Therefore, most patients present with pain and other symptoms.^(38,39)^

Stage 3 is characterized by exposed necrotic bone extending beyond the alveolar bone. In this final stage, bone tissue adjacent to the alveolar region may be involved, resulting in pathological fractures, extraoral fistulas, and oroantral communication.^(7,21)^

Although the AAOMS staging system remains the most widely adopted classification for MRONJ, it is primarily based on clinical presentation and the presence of exposed bone.^(7,38,39)^ However, accumulating evidence suggests that radiographic alterations may precede clinical bone exposure^(33,36,40)^ and facilitate earlier diagnosis while improving assessment of lesion extent. Accordingly, alternative classification systems, including those developed by the Italian Society of Oral Pathology and Medicine and the Italian Society of Maxillofacial Surgery (SIPMO-SICMF)^(33)^ incorporate both clinical and radiographic findings into disease staging.

These imaging-based approaches emphasize radiographic features, including osteosclerosis, cortical disruption, trabecular bone alterations, sequestration, sinus involvement, and the extent of bone involvement^(40)^ thereby providing a more comprehensive assessment of disease severity and progression. Therefore, integrating clinical and imaging findings may improve MRONJ diagnosis, staging, treatment planning, and longitudinal follow-up.^(7,33,37)^

### Prevention and treatment

All methods used to prevent or treat MRONJ focus on optimizing soft and bone tissue healing owing to the possible deleterious effects of antiresorptive and antiangiogenic drugs.

Because the etiopathogenesis of MRONJ remains incompletely understood, its treatment continues to be challenging. Clinical staging guides the selection of surgical or conservative treatment, either alone or in combination with adjuvant therapies.^(41-43)^ Khan et al.^(44)^ recommended that age, sex, systemic comorbidities, primary disease state, lesion size, exposure to antiresorptive drugs, and disease stage should all be considered when individualizing treatment.

Conservative treatment is generally indicated for patients in early stages of the disease or as an adjunct to surgical procedures. Common conservative measures include maintaining oral hygiene, using oral antiseptics, administering systemic antibiotics, and controlling periodontal diseases and other conditions that may predispose patients to MRONJ.^(37,41,43)^ Khan et al.^(44)^ also emphasized that conservative therapy forms the foundation of management and, though it may not achieve complete resolution, may improve prognosis and quality of life, which are the main objectives of treatment.

Surgical treatment includes sequestrectomy, which involves removing bone sequestra identified on imaging as exposed necrotic bone detached from the underlying basal bone, and local debridement to remove tissues involved in the lesion, including adjacent soft tissue (Figure 1). These procedures are generally restricted to patients with stage 2 or higher and those with severe symptoms.^(34,35)^ In a systematic review, Ruggiero et al.^(43)^ also reported that surgical intervention may be beneficial in patients with pain, even in the absence of bone sequestration.

**Figure 1:**
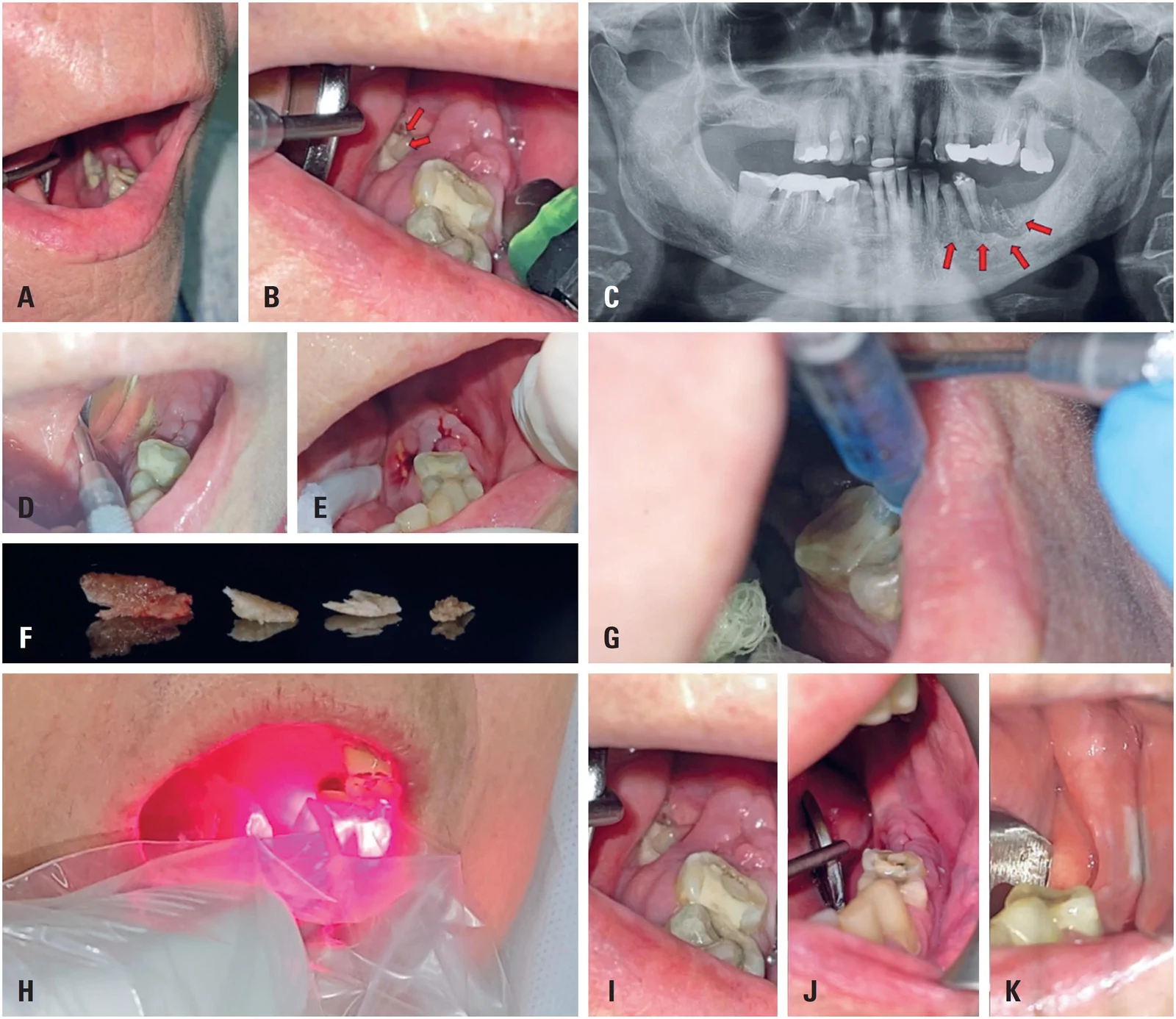
A) Clinical view, B) close-up view, and C) radiographic view of stage 3 medication-related osteonecrosis of the jaw affecting the left mandible before sequestrectomy; D) removal of the necrotic bone using piezoelectric bone surgery; E) surgical site after removal of the necrotic bone; F) removed bone fragments; G) before antimicrobial photodynamic therapy, 4mL of 100µg/mL methylene blue was applied for 1 min as a pre-irradiation step. H) Application of 660-nm red light to the posterior region of the right mandible (100mW; 6J/point); comparison of the treated area at I) baseline, J) 7 days after photodynamic therapy, and K) 60 days after treatment. Note the complete closure of the area

Symptomatic patients with pathological fractures and extensive necrotic bone tissue often require total or partial resection, even in isolated cases. This emphasizes the importance of an individualized treatment approach that considers the patient's symptoms, clinical history, and other relevant factors.^(42,43)^

While conservative and surgical strategies remain the foundation of MRONJ management^(7,43,44)^ evidence supporting most adjunctive therapies remains limited. Photodynamic therapy has shown promising results in preclinical and small clinical studies^(41,45)^ although evidence from large, randomized trials is lacking. Similarly, ozone therapy^(46,47)^ and platelet derivatives^(48-50)^ have demonstrated encouraging biological effects.

Additionally, adjuvant therapies have been proposed to enhance impaired tissue repair in these patients. Among these, antimicrobial photodynamic therapy has been the most extensively investigated in recent preclinical studies. It uses red or infrared light on areas pre-sensitized with photosensitizing solutions that are selectively absorbed by microbial cells, inducing cell death through hyperoxygenation.^(41,45)^ Ervolino et al.^(41)^ reported that this therapy enhanced bone repair and soft-tissue fibroblast activity while also exerting antimicrobial effects that may improve the local immune response and neoangiogenesis. These findings are consistent with those of Momesso et al.,^(45)^ who reported complete healing in 90% of patients treated with photodynamic therapy alone and 73.6% of those receiving combined surgical treatment.

Ozone therapy has also been considered a promising treatment at the tissue level because of its antimicrobial properties and potential to promote wound healing. It can be administered systemically or locally to prevent MRONJ or support its treatment.^(46-49)^ Xiao et al.^(46)^ and Ripamonti et al.^(47)^ reported favorable outcomes with local ozone therapy, including enhanced fibroblast migration, antimicrobial activity, and neoangiogenesis.

A recent systematic review and meta-analysis of six studies (178 patients) reported a pooled clinical success rate of 71% for adjunctive ozone therapy in MRONJ, with no reported adverse effects and frequent improvements in pain and quality of life. Nevertheless, the authors rated the certainty of evidence as very low and emphasized the need for randomized clinical trials with standardized treatment protocols.^(49)^

Fluorescence-guided surgery is an adjuvant modality that can assist surgical management by distinguishing necrotic from viable bone, facilitating local debridement while preserving healthy tissues. Before surgery, doxycycline, an antibiotic, is administered as a fluorescent bone marker. Under ultraviolet or blue light (400–460nm), collagen molecules exhibit autofluorescence, allowing identification of viable tissues. Necrotic bone can then be removed using drills under copious irrigation or piezoelectric surgery, minimizing damage to adjacent soft tissues.^(50)^

Another systemic adjuvant approach is the combined use of pentoxifylline and tocopherol for the treatment and prevention of MRONJ. Pentoxifylline, which is used to treat peripheral vascular diseases, improves blood flow by reducing erythrocyte viscosity and promoting fibrinolysis. Tocopherol (vitamin E) acts as an antioxidant, reducing oxidative stress and inflammation.^(8)^ Heifetz-Li et al.^(42)^ reported that combination therapy improved symptoms and promoted both hard and soft tissue healing with minimal adverse effects.

Platelet derivatives have been suggested as adjunctive local therapies for MRONJ.^(51,52)^ They are widely used in bone reconstruction because of their ability to modulate the local inflammatory response and promote chemotaxis of osteogenic cells while supporting soft tissue healing.^(18,19)^ Adornato et al.^(53)^ reported complete wound closure following surgical treatment combined with platelet derivatives, resulting in improved symptom control and quality of life.

Although further research is necessary to establish definitive and adjuvant treatment protocols for MRONJ, the therapies discussed have demonstrated promising results in relieving symptoms and improving patients’ quality of life. Combined therapeutic approaches may provide additional benefits, particularly in severe cases. However, preventing MRONJ remains a major challenge in the management of patients receiving antiresorptive or antiangiogenic therapy. ^(7,21)^

Complete healing of the necrotic lesion remains difficult to achieve and represents the ultimate goal of treatment. Although many therapies improve clinical symptoms and control local tissue inflammation, complete healing cannot be considered achieved until the necrotic area has fully resolved.

The search for therapies that restore normal bone remodeling remains one of the main challenges in MRONJ management. Recently, romosozumab, a monoclonal antibody approved for the treatment of osteoporosis in women at high risk of fracture, has emerged as a potential therapeutic alternative. This drug modulates the Wnt signaling pathway by inhibiting sclerostin, thereby increasing bone formation while simultaneously decreasing osteoclastic activity, resulting in a more physiological pattern of bone remodeling.^(7)^ Although the reported risk of MRONJ with romosozumab remains low (0.03–0.05%), it may represent an alternative treatment option for osteoporosis, especially in patients with light and moderate disease.^(28)^

Many patients still require potent antiresorptive drugs, which significantly increase the risk of MRONJ. Therefore, prevention remains the most effective strategy and should include regular monitoring from the initiation of antiresorptive therapy.

Recently, our group^(54)^ published a case series with long-term follow-up, providing relevant insights into the management of medication-related osteonecrosis of the jaw. In a prospective study involving 14 patients diagnosed with MRONJ at different stages (ranging from stage 0 to stage 3) and followed for up to 3 years, a multimodal therapeutic approach combining conservative surgical management with local and systemic adjuvant therapies was associated with favorable clinical outcomes. The proposed protocol comprised conservative sequestrectomy when indicated, antimicrobial photodynamic therapy, fluorescence-guided surgery for precise removal of necrotic bone, adjunctive use of platelet-rich fibrin, and supportive pharmacological therapy (Figure 2). Most patients achieved complete clinical remission, whereas the remaining patients demonstrated significant clinical improvement, characterized by pain reduction, decreased suppuration, and stabilization of exposed bone areas. Collectively, these findings suggest that, beyond surgical intervention alone, integrated therapeutic strategies targeting infection control, optimization of the local biological environment, and enhancement of tissue repair may improve clinical outcomes across different stages of MRONJ. In this context, adjuvant therapies, particularly antimicrobial photodynamic therapy and local antimicrobial approaches, have demonstrated satisfactory results in optimizing both bone tissue and soft tissue coverage and should be considered components of preventive and therapeutic strategies for MRONJ.^(55)^

**Figure 2:**
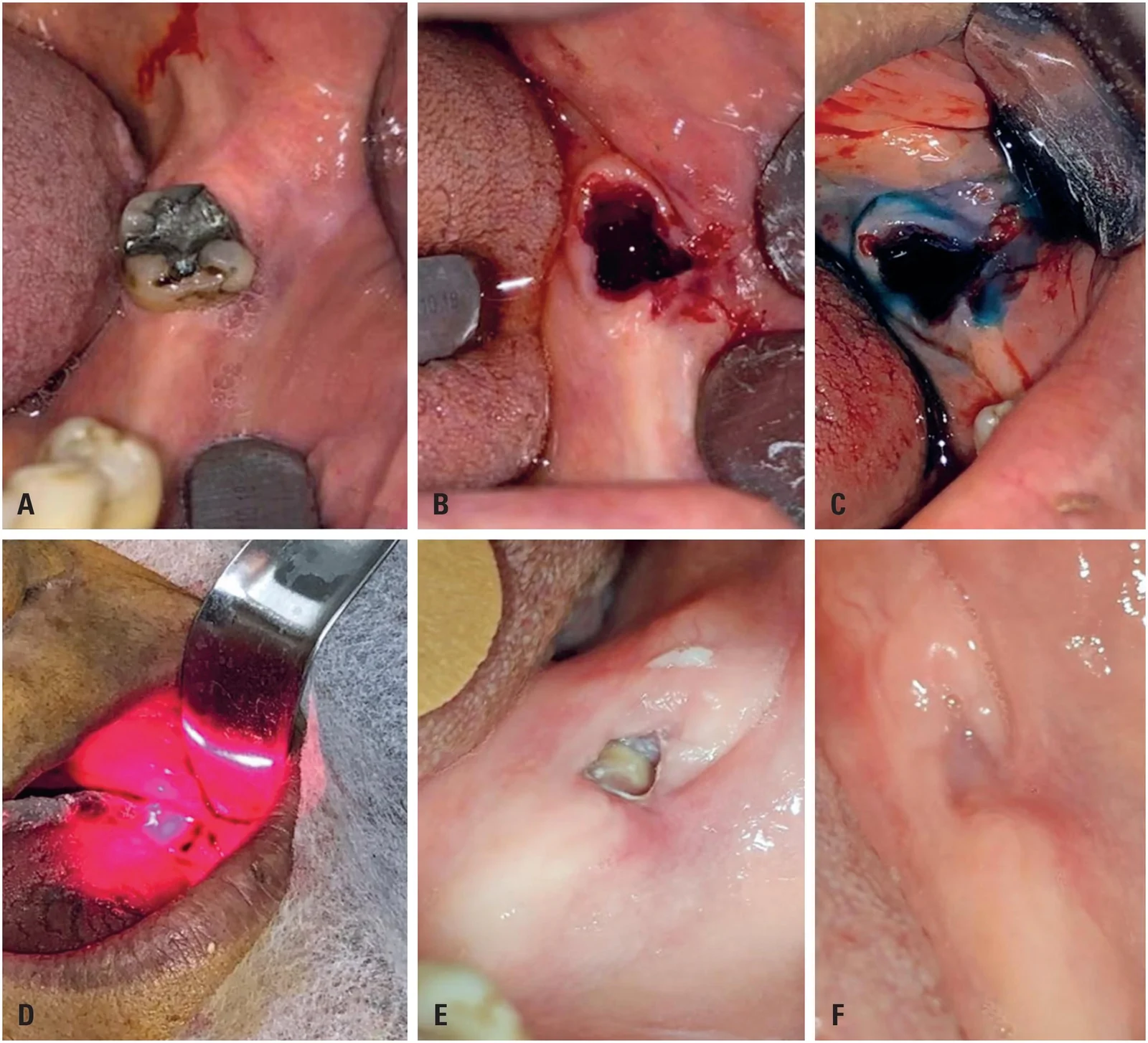
Clinical case illustrating the prevention of medication-related osteonecrosis of the jaw in a patient reporting pain in the left mandibular second molar using zoledronic acid every 6 months for 2 years for osteoporosis. (A) Initial clinical view of the tooth scheduled for extraction. B) Intraoperative view after tooth extraction. (C) Application of methylene blue as a pre-irradiation step for antimicrobial photodynamic therapy. Approximately 1mL of the solution was applied to the extraction socket (100µg/mL) for 1 min. (D) Application of 660nm red light to the socket (100mW; 6J/point). (E) Clinical outcome 2 weeks after tooth extraction. (F) Clinical outcome 8 weeks after surgery, showing complete healing with complete soft tissue covering

## CONCLUSION

Medication-related osteonecrosis of the jaw is a complex complication associated with antiresorptive and antiangiogenic therapies. Its pathogenesis involves impaired bone remodeling, vascular damage, inflammation, microbial colonization, and soft tissue deterioration. Current evidence indicates that the risk of medication-related osteonecrosis of the jaw varies according to the underlying disease and treatment plan, especially in patients with cancer. Clinical and imaging classification systems are crucial for guiding treatment. Although advances in conservative, surgical, and adjunct therapies have improved disease management and tissue repair, prevention and close monitoring of high-risk individuals remain fundamental to improving clinical outcomes. Future studies should prioritize the development of individualized risk prediction models and standardized treatment algorithms to optimize the prevention and management of medication-related osteonecrosis of the jaw across different patient populations.

## Data Availability

The content is already available.
